# Effect of Home-Based Telemonitoring Using Mobile Phone Technology on the Outcome of Heart Failure Patients After an Episode of Acute Decompensation: Randomized Controlled Trial

**DOI:** 10.2196/jmir.1252

**Published:** 2009-08-17

**Authors:** Daniel Scherr, Peter Kastner, Alexander Kollmann, Andreas Hallas, Johann Auer, Heinz Krappinger, Herwig Schuchlenz, Gerhard Stark, Wilhelm Grander, Gabriele Jakl, Guenter Schreier, Friedrich M Fruhwald, and the MOBITEL investigators

**Affiliations:** ^10^For a list of additional MOBITEL investigators see Acknowledgments; ^9^Department of CardiologyWilhelminenspital ViennaViennaAustria; ^8^Department of Internal MedicineHospital Hall/TirolHall/TirolAustria; ^7^Department of Internal MedicineHospital DeutschlandsbergDeutschlandsbergAustria; ^6^Department of CardiologyHospital Graz WestGrazAustria; ^5^Department of CardiologyHospital VillachVillachAustria; ^4^Department of CardiologyHospital WelsWelsAustria; ^3^Department of Internal MedicineHospital TullnTullnAustria; ^2^Information Management & eHealthAIT Austrian Institute of Technology GmbHGrazAustria; ^1^Department of CardiologyMedical University GrazGrazAustria

**Keywords:** Heart failure, telemedicine, mobile phone, eHealth

## Abstract

**Background:**

Telemonitoring of patients with chronic heart failure (CHF) is an emerging concept to detect early warning signs of impending acute decompensation in order to prevent hospitalization.

**Objective:**

The goal of the MOBIle TELemonitoring in Heart Failure Patients Study (MOBITEL) was to evaluate the impact of home-based telemonitoring using Internet and mobile phone technology on the outcome of heart failure patients after an episode of acute decompensation.

**Methods:**

Patients were randomly allocated to pharmacological treatment (control group) or to pharmacological treatment with telemedical surveillance for 6 months (tele group). Patients randomized into the tele group were equipped with mobile phone–based patient terminals for data acquisition and data transmission to the monitoring center. Study physicians had continuous access to the data via a secure Web portal. If transmitted values went outside individually adjustable borders, study physicians were sent an email alert. Primary endpoint was hospitalization for worsening CHF or death from cardiovascular cause.

**Results:**

The study was stopped after randomization of 120 patients (85 male, 35 female); median age was 66 years (IQR 62-72). The control group comprised 54 patients (39 male, 15 female) with a median age of 67 years (IQR 61-72), and the tele group included 54 patients (40 male, 14 female) with a median age of 65 years (IQR 62-72). There was no significant difference between groups with regard to baseline characteristics. Twelve tele group patients were unable to begin data transmission due to the inability of these patients to properly operate the mobile phone (“never beginners”). Four patients did not finish the study due to personal reasons. Intention-to-treat analysis at study end indicated that 18 control group patients (33%) reached the primary endpoint (1 death, 17 hospitalizations), compared with 11 tele group patients (17%, 0 deaths, 11 hospitalizations; relative risk reduction 50%, 95% CI 3-74%, *P* = .06). Per-protocol analysis revealed that 15% of tele group patients (0 deaths, 8 hospitalizations) reached the primary endpoint (relative risk reduction 54%, 95% CI 7-79%, *P*= .04). NYHA class improved by one class in tele group patients only (*P*< .001). Tele group patients who were hospitalized for worsening heart failure during the study had a significantly shorter length of stay (median 6.5 days, IQR 5.5-8.3) compared with control group patients (median 10.0 days, IQR 7.0-13.0; *P*= .04). The event rate of never beginners was not higher than the event rate of control group patients.

**Conclusions:**

Telemonitoring using mobile phones as patient terminals has the potential to reduce frequency and duration of heart failure hospitalizations. Providing elderly patients with an adequate user interface for daily data acquisition remains a challenging component of such a concept.

## Introduction

Chronic heart failure (CHF) is a major cause of hospitalization in Western countries, with a high rate of re-admission [1]. Despite effective therapies, patients with CHF suffer from a high rate of hospital re-admission and bear a high risk of death within the first months after an episode of acute heart failure [1,2].

Reduction of re-admissions is a major goal in the management of CHF patients. Therefore, both the European and the American guidelines on CHF recommend disease management programs that include telephone follow-up or home nursing [3,4].

Monitoring body weight, home visits by nurses, telephone conferences with nurses, and complete telemedical supervision have been tested with inconsistent results [5-20]. Simple interventions such as measuring body weight with telemedical surveillance produced conflicting results for re-hospitalization [5,8]. Nurse visits at home or telephone contact with nurses [6-7,10-12,14] inconsistently showed a benefit for the prognosis of heart failure patients.

Telemedicine using transmission of vital parameters can be expensive and technically demanding [12,14,21]. The majority of reports show a positive influence on the outcome of heart failure patients [9,12,15], although it is not clear which system is best for this challenge.

Rapid advances and the ubiquitous availability of mobile phones have created new perspectives toward telemedical interaction between patients and health care professionals [22]. Recently, we were able to show that mobile phone–based surveillance is safe and promising in cardiac patients [23].

Based on these promising early results using mobile phone technology, we designed the MOBITEL (MOBIle TELemonitoring in heart failure patients) study in order to test the hypothesis that telemedical surveillance using widespread mobile phone technology as patient terminals improves the outcome of CHF patients after an episode of acute cardiac decompensation.

## Methods

### Patients

MOBITEL was a prospective, randomized, open-label study. Recruitment started on October 1, 2003, and the study was closed with the sign-off of the last patient on April 29, 2008. Registration in a public trials registry was not performed for MOBITEL since it was optional at the time of study start. Local regulatory authorities approved the study, with the University Clinic Graz being the lead ethics committee.

 Patients were eligible for the study if they met all of the following inclusion criteria: acute worsening of heart failure (acute cardiac decompensation) with hospital admission lasting > 24 hours within the last 4 weeks, treatment according to the guidelines of the European Society of Cardiology (ESC) with an angiotensin converting enzyme (ACE) inhibitor or an angiotensin receptor blocker (ARB), diuretic, and beta-blocker (except in cases with documented intolerance to beta-blockers). Initially, patients older than 18 years and younger than 75 years were eligible; the latter was amended to 80 years after 4 months of recruitment. For the definition of CHF, we adopted the ESC guidelines [4].

Patients with one of the following conditions were not eligible for MOBITEL: unstable coronary artery disease with revascularization within the last 6 months, planned revascularization (percutaneous or surgical) for coronary artery disease, planned heart valve surgery, planned or completed heart transplantation, uncontrolled arterial hypertension, acute myocarditis, inability to read the display of a handheld phone, or malignancy.

After receiving verbal and written study information, patients gave written consent to participate in the study. Patients were allocated randomly to pharmacological treatment (control group) or pharmacological treatment plus telemedical surveillance (tele group). The adaptive randomization procedure was stratified by patient age, New York Heart Association (NYHA) class, gender, and study center.

Baseline demographics and medication were recorded for all patients, and an appointment for the 6-month follow-up was made. There was no planned interaction between study site and patients in the control group within the follow-up period of 6 months. Patients in the tele group were given the telemonitoring equipment and an appointment for telephone or face-to-face technical training.

### Equipment and Data Processing

The telemonitoring equipment consisted of three commercially available components: (1) a mobile phone (Nokia 3510, Finland), (2) a weight scale with 0.1 kg accuracy and electronic display (Soehnle creta, Germany), and (3) a sphygmomanometer for fully automated measurement of blood pressure and heart rate (BosoMedicus, Bosch&Sohn, Germany). Tele group patients were trained in measurement of blood pressure and weight using the equipment prior to discharge home. Furthermore, tele group patients were instructed by a study technician in the use of the mobile phone.

**Figure 1:**
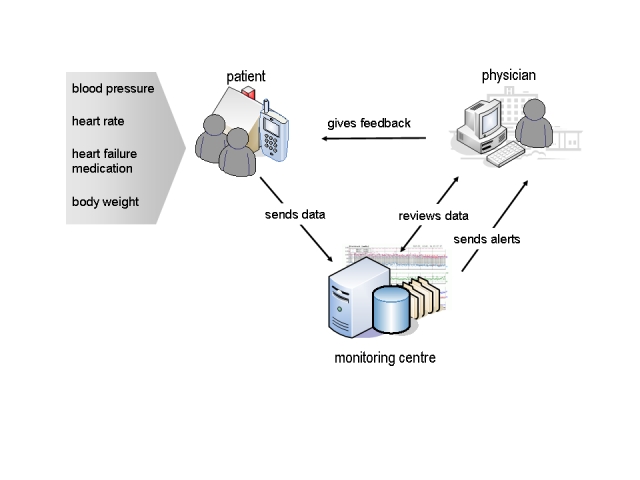
Schematic depiction of the equipment and data collection process used in MOBITEL

Tele group patients were asked to measure vital parameters (blood pressure, heart rate, body weight) on a daily basis at the same time, preferably in the morning after emptying the bladder and before dressing and taking medication. Thereafter, patients were advised to enter these values as well as their dosage of heart failure medication into the mobile phone’s Internet browser and send them to the monitoring center provided by the Austrian Institute of Technology (AIT) – Information Management & eHealth, Graz. Study physicians had access to a secure website providing both numerical and graphical depiction of data for each patient. Whenever necessary, study physicians could contact patients using the mobile phone. Figure 1 outlines this process.

**Figure 2:**
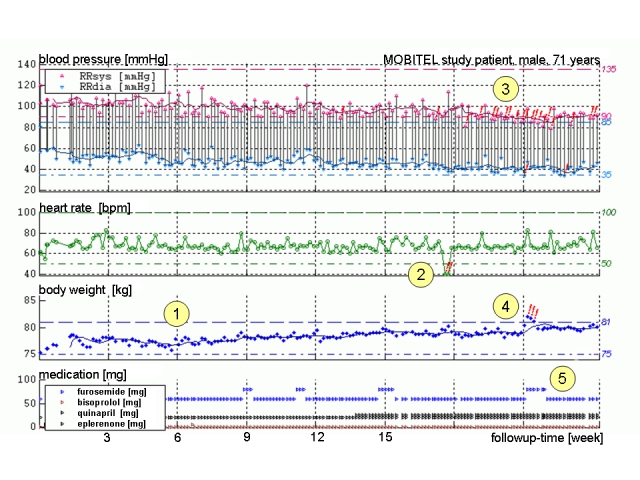
Trend chart of vital parameters of a typical patient (male, 71 years) over the 6-month study period: Each dot represents a transmitted value of (from top to bottom) systolic (red) and diastolic blood pressure (light blue), heart rate (green), body weight (blue), and heart failure medication (bottom panel, furosemide in blue); (1) After discharge from hospital, all transmitted values are stable, (2) Red exclamation marks indicate low heart rate, (3) Alarms due to decreased blood pressure herald a phase of instability, (4) Increase in body weight of more than 2 kg in 2 days, causing an alarm (red exclamation marks), (5) After telephone contact with the patient and confirmation of values, the daily dose of furosemide is temporarily increased and body weight returns to stable values within 3 days

At the monitoring center, data were depicted both numerically and graphically in an electronic case record form (CRF; Figure 2). Study physicians had continuous access to the CRFs of their patients via a secure website. Physicians were advised to use the automated warning system for the monitoring of vital parameters of their patients. If transmitted values went outside individually adjustable borders, study physicians were sent an email alert. Additionally, an email alert was generated if a patient’s body weight increased or decreased more than 2 kg in 2 days. After receiving an alert, study physicians could contact the patient directly via the mobile phone to confirm the parameters and, if appropriate, could ask the patient to adjust his or her medication.

For technical questions, patients had access to a 24-hour hotline at the service center.

### MOBITEL Telemedicine Platform

The MOBITEL telemedicine platform was developed as a three-tier, client-server architecture (data, logic, and representation layers) using state-of-the-art Internet technology. The Zope Web/application server (Zope 2.6.1, Zope Corporation, Fredericksburg, VA, USA) and the relational database system (Interbase 6.0, Borland Software Corporation, Cupertino, CA, USA) were chosen for the basic system.

While the application server provided core logic, particular services were developed as independent modules in the sense of service-oriented architecture (SOA). Particular functionalities were clustered into services that were able to communicate and share data with each other.

- data processing and graphic service: For sophisticated data processing and visualization of time-series data (e.g. blood pressure measurements), the MatLab 6.5 environment (The MathWorks, Natick, MA) was used.
- notification service: The database was checked at regular intervals for arrival of new alerts, notifications, or reminders generated by the data processing service. Subsequently, a personalized message was composed and sent to the responsible physician by text messaging, email, or both.

The components of the MOBITEL telemedicine platform were designed with respect to a high level of security and confidentiality to comply with regulatory requirements. Data transfer was encrypted, and access to the data was restricted to authorized users.

### Outcome Parameters and Endpoint Definitions

The combined primary endpoint of this study was cardiovascular mortality or re-hospitalization for worsening heart failure. Besides evaluation of patients’ functional status according to the NYHA classification and length of stay during re-hospitalizations, further secondary endpoints focused on technical parameters: system availability, cumulative transmissions, and transmissions per patient.

Study patients were classified as reaching the primary endpoint in case of hospital admission for worsening heart failure or cardiovascular death within the study period.

Tele group patients who dropped out immediately after randomization due to inability to handle the telemonitoring equipment despite intensified training were classified as “never beginners.” These patients were contacted 6 months after randomization to obtain data for the primary endpoint.

Study patients who stopped prematurely for personal reasons were classified as “early termination on patient’s request.” These patients were invited to an earlier follow-up (i.e. end-of-study visit) to obtain as much information as possible and were included in the final analysis according to randomization.

### Statistics

For statistical planning, we assumed that patients in the control arm would show an event rate of 30% over 6 months [1]. For the telemedicine arm, we expected a 50% reduction of the event rate. To show a statistically significant difference at an error of .05 with a power of 80%, a sample size of 240 subjects was calculated.

Randomization was stopped after 120 patients due to an increasing number of never beginners who were unable to operate the mobile phone, indicating the urgent need for a new technology. However, as we tried to avoid a mix of technologies within one study, we decided to stop randomization in coordination with the ethics committee of the Medical University Graz. Therefore, the results must be interpreted cautiously.

Final data analysis was performed according to the per-protocol principle, including all patients except never beginners. Additionally, intention-to-treat analysis was performed, including all randomized patients. The log-rank test, the Kaplan-Meier estimation method with 95% confidence interval (CI), and calculation of the relative risk reduction were used to analyze the primary endpoint.

With respect to the secondary endpoints, difference in functional NYHA class between baseline and end of study was compared between patients in the control and tele group. Dosage of heart failure medication was calculated as percentage of the ESC-recommended dosage and compared between both groups.

Normally distributed values were compared using the t-test, while the chi-square test was used to compare nominal distributed values. We used the Wilcoxon rank sum test to compare independent samples and the Wilcoxon signed rank test to compare dependent samples when values were not normally distributed.

Patients’ adherence with the telemonitoring system was expressed as a percentage of effectively to expected received datasets. Thus, the cumulative monitoring period and the total number of received values were calculated for all participants.

Statistics were calculated using R statistical software, version 2.4.1 (R Foundation for Statistical Computing, Vienna, Austria). We considered a P-value < .05 to show a statistically significant difference in all comparisons. All data are given as median and interquartile range (IQR).

## Results

### Patients

**Figure 3:**
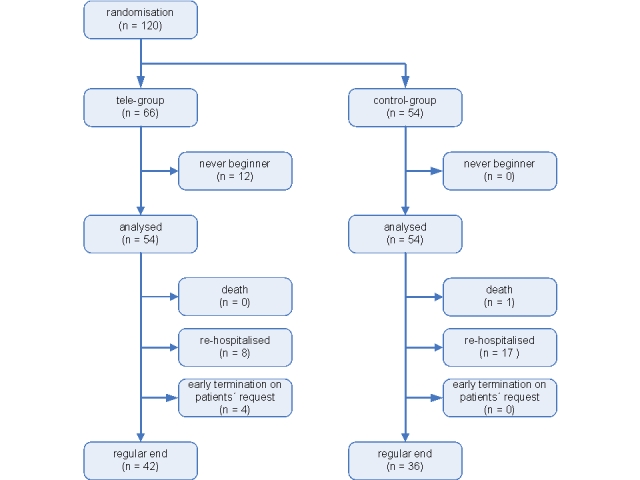
Study flowchart of the MOBITEL population

In the tele group, 12 patients (6 male, 6 female; median age 68 years [IQR 64-74]) emerged unable to begin transmission of data and were classified as never beginners. Never beginners were included in the intention-to-treat analysis but not the per-protocol analysis. Furthermore, there were four patients who requested early termination of the study (4 male, median age 63 years [IQR 60-65]) and were included in both the intention-to-treat analysis and the per-protocol analysis. Figure 3 shows the study flowchart.

Overall, 120 patients (85 male, 35 female) with a median age of 66 years (IQR 62-72) were randomized in eight centers: 54 patients were randomized to the control group (39 male, 15 female; median age 67 years [IQR 61-72]), and 66 patients were randomized to the tele group (46 male, 20 female; median age 66 years [IQR 62-73]).

For the per-protocol analysis (Table 1), the control group comprised 54 patients (39 male, 15 female; median age 67 years [IQR 61-72]) and was compared with the tele group, including 54 patients (40 male, 14 female; median age 65 years [IQR 62-72]). At baseline there was no statistically significant difference between the two groups regarding age, gender, cause of heart failure, NYHA functional class, left ventricular (LV) ejection fraction, heart failure medication, as well as frequency and duration of heart failure hospitalizations in the year prior to randomization (Table 1).

**Table 1:** Baseline characteristics of the MOBITEL population (per-protocol population)

Characteristic	Control Group (n = 54)	Tele Group (n = 54)	*P* (for difference)
Median age, years (IQR)	67 (61-72)	65 (62-72)	.79
Male, no. (%)	39 (72)	40 (74)	.92
Median LV ejection fraction (IQR)	29 (21-36)	25 (20-38)	.70
NYHA class II, no. (%)	7 (13)	7 (13)	.40
NYHA class III, no. (%)	37 (68.5)	33 (61)	.40
NYHA class IV, no. (%)	10 (18.5)	14 (26)	.40
Median number of HF^a^ hospitalizations in past 12 months, no. (IQR)	1 (1-2)	1 (1-2)	.36
Median length of stay for HF hospitalizations, days (IQR)	11 (7-17)	12 (9-15)	.67
**Cardiovascular Risk Profile**			
Ischemic heart disease, no. (%)	23 (43)	20 (37)	.69
Hypertension, no. (%)	24 (44)	29 (54)	.44
Valvular disease, no. (%)	1 (2)	1 (2)	.48
Diabetes mellitus, no. (%)	16 (30)	12 (22)	.44
**HF Treatment at Study Entry**			
ACE inhibitor, no. (%)	41 (76)	45 (83)	.28
ARB, no. (%)	13 (24)	9 (17)	.47
Diuretic, no. (%)	44 (81)	49 (91)	.27
Beta-blocker, no. (%)	42 (78)	47 (87)	.31
Spironolactone, no. (%)	23 (43)	21 (39)	.85

^a^HF = heart failure.

**Figure 4:**
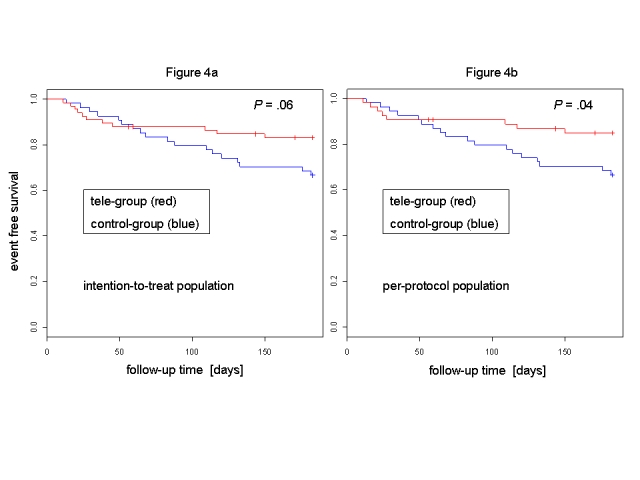
Kaplan-Meier curve for primary endpoint in MOBITEL: intention-to-treat analysis (4a) and per-protocol analysis (4b)

Intention-to-treat analysis indicated that 18 control group patients (33%) reached the primary endpoint (1 death, 17 hospitalizations) compared with 11 tele group patients (17%, 0 deaths, 11 hospitalizations; relative risk reduction 50%, 95% CI 3-74%, *P*= .06; Figure 4a). The number of never beginners reaching the primary endpoint was not higher than for control group patients.

### Per-Protocol Analyses

Per-protocol analysis at study end revealed that 15% of tele group patients (0 deaths, 8 hospitalizations) reached the primary endpoint (relative risk reduction 54%, 95% CI 7-79%, *P*= .04; Figure 4b).

Median NYHA class improved from 3 to 2 in tele group patients only (*P*< .001) compared with control group patients at study end and compared with tele group baseline values. Ejection fraction showed a nonsignificant improvement in both the control group, from 29% (IQR 21-36) to 35% (IQR 24-40), and the tele group, from 25% (IQR 20-38) to 35% (IQR 25-45).

Tele group patients who were hospitalized for worsening heart failure during the study had a significantly shorter length of hospital stay (median 6.5 days, IQR 5.5-8.3) compared with control group patients (median 10.0 days, IQR 7.0-13.0; *P*= .04).

Dosage of heart failure medication was calculated as percent of the ESC-recommended daily dose. At baseline, tele group patients had a higher percentage of the ESC-recommended daily dose of ACE inhibitors compared to control group patients. However, this difference was no longer significant at study end. All other types of drugs were equally balanced.

Tele group patients started transmission of data within 6.5 days (IQR 4-11) after randomization. During the entire study, a total of 7554 value sets were received from tele group patients, corresponding to a median of 162 transmissions per patient (IQR 136-173). On 7554 out of 7962 cumulative monitoring days, at least one set of values was sent, which indicates a patient adherence rate of 95%.

There were 375 alerts sent to study physicians in cases of exceeding predefined thresholds for body weight or exceeding the dynamic threshold of ± 2 kg in 2 days. Consequently, tele group patients were contacted 170 times. In 55 of those times, an adjustment of heart failure medication was made (Table 2). Adverse events or side effects were not observed.

**Table 2:** Technical data and interventions in the telemedicine arm

Variable	Tele Group (n = 54)
System availability, %	98
Patient adherence, %	95
Cumulative monitoring, days	7962
Cumulative transmissions, no.	7554
Median transmissions/patient, no. (IQR)	162 (136-173)
Patient contacts following alarms, no.	170
Adjustments of individual limits, no.	118
Adjustments of medication, no.	55

## Discussion

The MOBITEL study addressed important issues in the management of heart failure patients after discharge from hospital, namely the following:

- how to reduce the high risk of re-hospitalization for worsening heart failure and/or cardiovascular death
- how to detect early warning signs of impending decompensation
- how to inform the treating physician

For this purpose, mobile phone–based patients terminals were used in a telemonitoring scenario. The system was developed and tested in 120 heart failure patients. After 6 months, there was a reduction of relative risk in tele group patients (re-admission or death) by 54%. However, the small sample size and the premature termination of randomization because of relevant technological issues with the patient terminal have to be considered as the main limitations when interpreting the results.

### Telemonitoring and Telemedical Intervention for Patients With Heart Failure

Patients suffering an episode of acute heart failure are at a high risk of adverse events within the first months after such an episode [1,2]. Therefore, efforts are needed to reduce this risk and to look out for early warning signs of acute heart failure.

Telemonitoring in general may provide a powerful tool to look for early warning signs and to reduce the high risk of adverse events in patients with CHF [19]. However, there are some uncertainties about the parameters to monitor as well as the proper monitoring tool.

The simple concept of monitoring body weight with telemedical surveillance did not show consistent results [5,8] but led to more sophisticated monitoring tools. However, recently it has been shown that the risk for re-hospitalization for worsening heart failure is highest in patients with weight gain of more than 4.5 kg within the week prior to hospitalization [24]. In our study, we also used increasing body weight as an early warning sign of fluid retention, although our limits were much closer (2 kg weight gain within 2 days). This stringent limit led to a significant number of alerts prompting communication between study physicians and patients, and, in some but not all cases, led to an adjustment of medication.

Increase in body weight often signals worsening heart failure. In addition, changes in blood pressure and heart rate may also herald worsening heart failure. To detect such episodes, we used a fully automated sphygmomanometer to measure both blood pressure and heart rate to gain information about potential refinement of heart failure therapy.

Our study is also the first to investigate the ability of patients to transmit information on heart failure medication dose on a daily basis. The vast majority of patients were able to enter and transmit their daily doses together with vital parameters by using the mobile Internet browser.

Furthermore, this study offered the unique ability to see that patients with CHF underwent an educational process: In many tele group patients who entered the study with some instability and who were (in response to alarms) advised by the study physician to adapt diuretic therapy, we saw, over time, some form of “self-monitoring” and, consequently, self-treatment.

As indicated by the late separation of Kaplan-Meier curves in Figure 4, telemedical surveillance does not appear to be superior to conventional treatment in the first month of follow-up. In our study, the majority of re-hospitalizations of tele group patients was seen in the first month of patient follow-up.

### Alarms and Alarm Management

In this study, sophisticated pre-analysis of data was not implemented. False-positive alarm messages sent to the physicians were predominately influenced by two factors:

- Outlier and type errors were not identified.
- Individual thresholds were not quickly adjusted in response to the slight variation of the body weight over time.

However, alarm management in general (i.e. timely confirmation of an alarm) and alarm generation in particular turned out to be crucial factors in a home-monitoring scenario. Hence, future research will focus on the development of sophisticated algorithms for automatic data analysis of  home-monitoring data. The algorithm should provide high sensitivity and specificity in order to make telemedical services effective and efficient.

### Mobile Phone–Based Patient Terminal

MOBITEL was the first study showing a positive influence on outcome in CHF patients using mobile phones as patient terminals for daily data transmission to the monitoring center.

Mobile phone technology (either GSM or 3G) is widespread in Europe and is a familiar tool, even in elderly patients. Hence, after 4 months of recruitment, we amended the upper age limit for inclusion up to 80 years.

The approach of using the mobile phone as telemonitoring equipment is different from all other studies reported so far: previous trials used more sophisticated equipment to obtain and transmit vital parameters from heart failure patients [9,12,15,21].

As the 95% patient adherence to the telemonitoring system indicates, the system was well accepted by those who were able to handle the mobile phone. Contrary to our expectations, data entry errors were rare, and the quality of self-reported data was appropriate for further clinical evaluation.

Recently, our group reported the first use of mobile phone–based telemedical surveillance in cardiac patients [23]. With this trial we were able to show that this technology can be used to monitor heart failure patients and to improve their outcome.

### Limitations

Not surprisingly, our study has a number of limitations. Although we were careful not to include patients with visual impairment (this was an explicit exclusion criterion), there were some patients who were not able to properly handle the mobile phone’s Internet browser (never beginners). However, there was no difference at baseline between these never beginners and the remaining study patients, and the never beginners subsequently had an event rate that was not different from control patients.

As a consequence, we looked for new technologies to improve the usability for elderly, technically unskilled patients. A promising solution could be the use of near field communication (NFC) in telemonitoring technology. Unlike Bluetooth, NFC supports a touch-based method for data acquisition using upcoming NFC-enabled mobile phones [24]. In addition to data acquisition from medical devices, it provides access to data stored on radio frequency identification (RFID) tags (eg, electronic barcodes), which could be embedded in future telemonitoring technology. However, as we did not want to mix two completely different technologies in one study, we decided to stop randomization after 120 patients. Therefore, the intention-to-treat analysis only revealed a trend in favor of telemedical surveillance, while the per-protocol analysis showed a significant difference between groups. This is the second major limitation of MOBITEL.

### Lessons Learned

Based on the experiences from the MOBITEL study, the following key requirements for the utilization of a telemonitoring system in CHF management have been identified:

- an easy-to-use patient terminal to allow safe and secure data acquisition, especially for unskilled, elderly patients
- sophisticated alarm management with high sensitivity and specificity in order to focus physicians’ attention on those patients whose data indicate signs of worsening health status

The development of an integrated care concept might be helpful to allow optimal integration of telemonitoring tasks into the existing workflows and processes of clinicians, general practitioners, and home care nurses.

### Conclusion

The results of the MOBITEL study indicate that home-based telemonitoring using mobile phones improves outcome in CHF patients and reduces both frequency and duration of heart failure hospitalizations. Providing elderly patients with an adequate user interface for data acquisition and transmission remains a challenging part of this concept.

## Abbreviations

ACE: angiotensin converting enzyme
ARB: angiotensin receptor blocker
CHF: chronic heart failure
CRF: case report form
ESC: European Society of Cardiology
IQR: interquartile range
LV: left ventricular
MOBITEL: MOBIle TELemonitoring in heart failure patients
NFC: near field communication
NYHA: New York Heart Association
